# Individualized functional magnetic resonance imaging neuromodulation enhances visuospatial perception: a proof-of-concept study

**DOI:** 10.1098/rstb.2023.0083

**Published:** 2024-10-21

**Authors:** Anthony Allam, Vincent Allam, Sandy Reddy, Eric M. Rohren, Sameer A. Sheth, Emmanouil Froudarakis, T. Dorina Papageorgiou

**Affiliations:** ^1^ School of Medicine, Baylor College of Medicine, Houston, TX, USA; ^2^ Department of Computer Science, University of Texas at Austin, Austin, TX, USA; ^3^ Dan L. Duncan Comprehensive Cancer Center, Baylor College of Medicine, Houston, TX, USA; ^4^ Department of Radiology, Baylor College of Medicine, Houston, TX, USA; ^5^ Department of Electrical and Computer Engineering, Rice University, Houston, TX, USA; ^6^ Department of Neurosurgery, Baylor College of Medicine, Houston, TX, USA; ^7^ Department of Basic Sciences, Medical School, University of Crete, Heraklion, Greece; ^8^ Institute of Molecular Biology and Biotechnology, Foundation for Research and Technology Hellas, Heraklion, Greece; ^9^ Department of Psychiatry, Baylor College of Medicine, Houston, TX, USA; ^10^ Department of Physical Medicine & Rehabilitation, Baylor College of Medicine, Houston, TX, USA; ^11^ Center for Space Medicine, Baylor College of Medicine, Houston, TX, USA; ^12^ Department of Neuroscience, Baylor College of Medicine, Houston, USA

**Keywords:** cortical blindness, mechanisms and methods of individualized neuromodulation, visuospatial perception and cognition

## Abstract

This proof-of-concept study uses individualized functional magnetic resonance imaging neuromodulation (iNM) to explore the mechanisms that enhance BOLD signals in visuospatial perception (VP) networks that are crucial for navigation. Healthy participants (*n* = 8) performed a VP up- and down-direction discrimination task at full and subthreshold coherence through peripheral vision, and superimposed direction through visual imagery (VI) at central space under iNM and control conditions. iNM targets individualized anatomical and functional middle- and medial-superior temporal (MST) networks that control VP. We found that iNM engaged selective exteroceptive and interoceptive attention (SEIA) and motor planning (MP) networks. Specifically, iNM increased overall: (i) area under the curve of the BOLD magnitude: 100% in VP (but decreased for weak coherences), 21–47% in VI, 26–59% in MP and 48–76% in SEIA through encoding; and (ii) classification performance for each direction, coherence and network through decoding, predicting stimuli from brain maps. Our findings, derived from encoding and decoding models, suggest that mechanisms induced by iNM are causally linked in enhancing visuospatial networks and demonstrate iNM as a feasibility treatment for low-vision patients with cortical blindness or visuospatial impairments that precede cognitive decline.

This article is part of the theme issue ‘Neurofeedback: new territories and neurocognitive mechanisms of endogenous neuromodulation’.

## 1. Introduction

Neuromodulation is a rapidly expanding precision-medicine approach that includes a spectrum of invasive and non-invasive interventions ranging from invasive deep brain stimulation to non-invasive, such as functional magnetic resonance imaging (fMRI) neurofeedback. Each of these interventions modulate neural activity, and can be evaluated as a treatment for psychiatric, neurological and general pathophysiological conditions.

**Figure 1.**
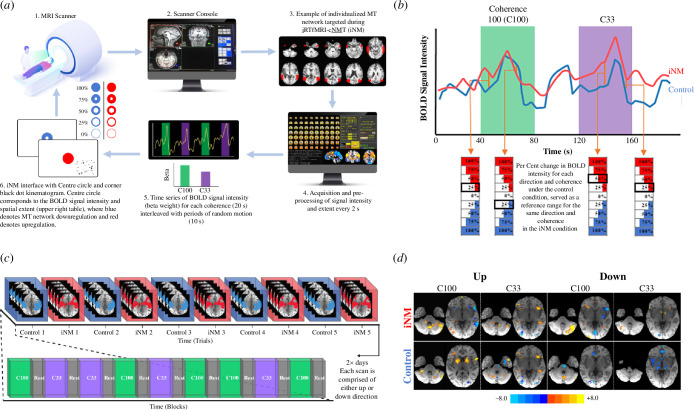
Individualized real-time fMRI closed-loop neuromodulation (iNM) intervention. (*a*) iNM strengthens visuospatial perception (VP) and visual imagery (VI) networks: (1) and (2) high-resolution anatomical images were acquired and registered to the Siemens’ console computer; (3) each participant’s individualized MT and MST networks were delineated and contoured (electronic supplementary material, figure SI–10); (4) iNM data extracted from individualized networks (electronic supplementary material, figure S10) was preprocessed and general linear modelling (GLM) decoded each coherence level in real-time every time repetition (TR) = 2 s (yellow line denotes the time series during green, purple and black periods representing up or down direction at coherence level C100, C33 and baseline-random motion period, respectively); (5) the BOLD signal intensity and spatial extent (individualized network) for each direction was computed through GLM, and beta weights were updated, see (*c*); (6) iNM interface shows the extent of the circle filled, which directly corresponds to the per cent upregulation (red fill) or downregulation (blue fill) of the BOLD signal intensity and spatial extent. Dots in the lower right corner of the iNM interface show the dot kinematogram. Dots travelled upwards or downwards at 100% or 33% coherence.(*b*) Computation of the iNM signal: the neuromodulation stimulus was calculated as the per cent change in BOLD signal intensity for each direction and coherence compared to the control run, which served as the reference for the same direction and coherence in the iNM condition (details under §2(g)). The following examples illustrate various scenarios of per cent signal change in BOLD intensity in the iNM condition: the first arrow indicates 25% increase in BOLD signal intensity compared to the control. The second arrow shows 25% decrease in BOLD signal intensity relative to the control for upward motion at 100% coherence. The next arrow points to 50% increase in BOLD intensity compared to the control, while the final arrow indicates 25% increase for upward motion at 33% coherence. Similar patterns were observed for downward motion at 100% and 33% coherence. (*c*) Task design (details in the electronic supplementary material, figure SI–2: the study was performed over 2 days in which five control and five iNM runs were completed in an alternating fashion. On the first day, we localized each subject’s MT and MST areas. On the second day, we targeted individualized MT/MST networks using iNM. Each run was performed in a temporal sequence that included: a coherent motion block lasting 20 s interleaved with a 10 s baseline-random motion block. The coherence level was counterbalanced across runs and blocks to ensure that the same coherence levels were not stacked back-to-back. (*d*) Brain activation maps show increased activation in the iNM condition compared to control: GLM-generated activation maps for each coherence level and motion direction in iNM (top) and control—no iNM (bottom) conditions are shown; voxelwise *p*-value = 0.05, cluster-size >20 voxels.

In this proof-of-concept study, we developed and tested the feasibility of a non-invasive, guided, individualized fMRI closed-loop neuromodulation (iNM; figure 1*a*) approach. This approach builds upon the principles of fMRI neurofeedback by offering a precision-medicine strategy that is uniquely tailored to each individual, specifically targeting neurological conditions. The long-term goal of our iNM approach is to neurorehabilitate cerebral visual impairment (CVI; [1]), specifically visuospatial perception (VP; [2,3]). CVI refers to damage in retrochiasmatic pathways and surrounding cortical visual areas that present with visual deficits, such as perception of complex motion, characterized by difficulty navigating the environment [4]. Specifically, two conditions associated with CVI can be targeted using iNM, for which neither effective, non-invasive and individualized treatments currently exist: (i) neurorehabilitation of VP deficits caused by cortical blindness; and (ii) deceleration of VP impairment, as a prodromal phase to cognitive impairment.

- Cortical blindness refers to visual loss owing to lesions along retrochiasmal pathways that originate below the optic chiasm to the visual cortex. Such lesions produce visual field deficits, including hemianopic or quadrantanopic scotomas [5–7] that impede the ability to detect objects within the central visual space [8]. Aetiological factors of cortical blindness most commonly include stroke (posterior and middle cerebral artery infarct) [9–11], head injuries and surgically induced embolisms [5,12–14]. Cortical blindness severely impacts patients' quality of life and has significant economic consequences [15–20]. Unfortunately, no current approaches effectively restore vision in these patients [21]. Behavioural attempts to restore vision, such as compensatory eye movements and visual restoration training do not have practical benefits to patients’ vision [22–32]. In a review of 13 visual field defect treatment studies, only six compared treatment with a control, and of those, only four (67%) showed minimal improvement in reading but not in other tasks of daily living, with no reduction in scotoma size [11]. Thus, no effective treatment is currently available to restore cortical blindness deficits.
- Subjective cognitive impairment (SCI)*,* a prodromal phase of mild cognitive impairment (MCI) leading to Alzheimer’s disease [33], is characterized by cognitive decline without objective deficits upon neuropsychological testing or other neurological or psychiatric diagnoses [34–39]. Since **visuospatial** deficits can precede memory impairment in very early phases of cognitive decline [39], SCI patients may benefit from VP rehabilitation. SCI affects 11.2% of adults aged over 45 years, with over half reporting functional limitations, and it is associated with a 1.5- to 3-fold increased risk of developing MCI or dementia [40,41]. SCI diagnostic work-up includes low vision impairment questions, defined as peripheral and central visual fields deficits. SCI diagnostic work-ups include questions on peripheral and central visual fields deficits referred to as low-vision impairment. Of adults aged over 45 years who self-reported vision impairment, 18% also reported SCI-related functional limitations, while only 4% of those without vision impairment reported similar limitations [42]. VP deficits that prevent scanning or navigating the environment (e.g. reading, catching a ball, walking and driving), or switching between visual tasks, can result from attention and memory deficits [39,42–45]. Early use of iNM to target the VP network in SCI has the potential to: (i) diagnose visuospatial impairment, since standard neuropsychological testing is not sensitive enough to identify these deficits; and (ii) decrease the rate of visuospatial deterioration, owing to a lack of interventions that slow functional cortical visual changes.

### (a) Visual processing and the visuospatial perception network

**Visual processing** begins with the detection of light by photoreceptors (rods and cones) in the retina, where visual information is converted into electrical signals. These signals travel through the optic nerve, optic chiasm and optic tract to the lateral geniculate nucleus (LGN) of the thalamus. The LGN serves as a critical relay station, transmitting this visual information to the primary visual cortex (V1), where initial cortical processing—visual awareness—takes place [46]. Beyond V1, visual information is further processed in higher-order visual areas [47]. This processing occurs through two distinct pathways that contribute to the interpretation and integration of visual data, ultimately forming a coherent perception of the environment: (i) the **dorsal stream** (the ‘where’ pathway) projects to the parietal lobe and is responsible for spatial processing, motion detection and guiding motor actions [48,49]; and (ii) the **ventral stream** (the ‘what’ pathway) projects to the temporal lobe and is involved in object recognition, including identifying shapes, colours and faces [50].

The VP network integrates information from the dorsal and ventral pathways to support a broad range of visual functions, primarily spatial awareness and motion detection, contributing to comprehensive visual processing in the environment [51]. It is more closely associated with the **dorsal stream**, as it supports essential visuospatial tasks, such as detecting and interpreting the movement of objects, spatial orientation (understanding spatial relationships between objects), navigation (using visual cues to move through space [48,50,52]) and visual attention (directing focus to salient stimuli, including areas that control eye movements [53]).

Motion detection structures that comprise the VP pathway *beyond key structures as the LGN to V1, V2, V3 (Brodmann areas 17, 18, 19*): (i) the ***middle temporal (MT/V5) area*** responsible for motion detection and interpreting the speed and direction of moving objects [50,51,54–62]; (ii) the ***medial-superior temporal (MST) area***, which processes complex motion patterns, such as optic flow and perception of object movement relative to the observer’s position [58,63–66]; and (iii) the **posterior parietal cortex (PPC)**, which creates cognitive maps of the environment by integrating sensory inputs and attention to guide motor actions [67–71].

### (b) The visuospatial perception and visual imagery networks for motion perception control

Because the long-term goal of our study is to address deficits resulting from low vision, which may affect peripheral, central or both visual fields following cortical blindness, and to slow the progression of VP impairments—prodromal symptoms of SCI—we designed a task that engages both central and peripheral vision. Since the brain relies on similar networks for both visual imagery (VI) and VP of motion, VI can enhance motion processing, particularly when direct visual input is compromised. This is accomplished by integrating spatial and contextual information, which is critical for navigation, planning and other complex cognitive functions [72,73].

Motion was presented in the peripheral visual field, which was detected and discriminated through activation of the VP network. In the central visual field (centre of the screen), which displayed coloured feedback with no motion, the task required subjects to mentally superimpose up or down direction of motion through the VI network, while simultaneously tracking up or down motion through their peripheral vision. Thus, this dual engagement activated both the VP and VI networks, which are supported by overlapping areas individualized middle temporal (MT/V5) area, medial superior temporal (MST), and posterior parietal cortex (PPC) - anatomical and functional networks for each participant for Up direction at 100% coherence, 33% of coherence, and Down direction at 100% coherence and 33% coherence; *p*-value < 0.05 [74,75]. The MT [58,61,75,76] and MST [59,65,66] areas are integral to both the VP and VI networks, processing not only actual motion perception but also motion imagery and spatial transformations. The PPC is crucial for spatial awareness, integrating sensory information in response to motion [69,77,78], both during actual motion perception and visual motion imagery tasks [70,72,79].

*By incorporating VI to enhance the VP-driven task, we engaged shared neural pathways also activated during actual perception* [75,76,79]. Engaging both central and peripheral visual spaces provided a more robust mechanism for detecting motion, with our future goal to retrain motion processing for the neurorehabilitation of cortical blindness or to maintain motion processing in SCI. Our feasibility study simulated a real-world ecosystem, as VI can strengthen VP-overlapping pathways, which are often partially lesioned or underused in disease states. VI strengthens the brain’s VP network by mimicking real-life challenges, using motor planning (MP) and selective attention networks, which are crucial for visuospatial and navigation abilities [67,80]. Our approach is supported by Farah [73], who emphasized how VI activates and strengthens neural pathways involved in visuospatial processing, providing a tool for reinforcing spatial cognition and enhancing the brain’s ability to process visuospatial tasks. By repeatedly engaging in these imagery-based processes, individuals can strengthen associative pathways in the brain, enhancing their ability to perform visuospatial tasks in real-life situations [81]. This aligns with [82], who advocate for fMRI interfaces that mirror real-world scenarios where deficits manifest. By simulating environments that provoke dysfunctional neural mechanisms, our neuromodulation interface aims to recruit and modify impaired brain functions [72].

### (c) Principles of individualized neuromodulation

The foundation of iNM begins with the precise localization of brain regions using localizer scans that identify areas with the highest average BOLD (the ratio of oxygenated to deoxygenated haemoglobin) signal intensity, as identified through GLM analysis (*p*‐value < 0.05; details under §2(c)). These regions are then targeted within the iNM protocol to modulate (upregulate or downregulate) new functional pathways that can then perform the impaired functions. In patients, this localization of regions with maximal BOLD intensity occurs in intact regions, **using the brain’s inherent functional redundancy**—referred to as functional copies.

To elaborate, following a lesion, brain regions that previously played the main role to perform visual and cognitive tasks can no longer support this role. iNM is based on using neural plasticity to bypass lesioned visual pathways that can no longer perform visual and cognitive tasks by training intact brain regions to perform these functions. iNM aims to induce changes by boosting supportive brain regions and/or ‘islands’ of intact tissue with the goal to at least partially fulfil the brain function previously led by the now-damaged areas. Thus, iNM uses the brain’s inherent redundancy to modulate the BOLD signal intensity and its spatial extents. We use these functional copies to pair individualized anatomical and functional networks (electronic supplementary material, figure SI-10) with targeted iNM training to enhance the physiological response. In the absence of an intact VP pathway, brain regions can establish new connections with intact visual areas by bypassing damaged pathways [83–87]. Thus, if early visual areas are lesioned, iNM can strengthen new areas (e.g. higher visual and/or higher cognitive areas) to perform the function once performed by the currently lesioned areas.

Functional redundancy in both healthy and disease state can be harnessed with targeted and guided training by aligning the following key factors:

- *individualized training*: focusing on MT, MST and middle occipital anatomical and functional areas (electronic supplementary material, figure SI-10). Individual brains vary substantially in cytoarchitectonic and macrostructural anatomy, and in their functional organization relative to their structural anatomy [88–90]. Although transcranial magnetic stimulation and transcranial direct current stimulation lack the precision of fMRI to target specific voxels down to 1 mm, both have shown that personalized targeting based on individual brain maps can optimize therapeutic effects, particularly in neurological and psychiatric disorders [91,92]. Therefore, developing approaches that account for variability in individual brain anatomy and function will significantly improve the efficacy of neuromodulation treatments, leading to better symptom management and patient quality of life [93]. With iNM, we use each participant’s unique structural and functional network to not only improve therapeutic outcomes for various neurological and psychiatric disorders but also to enhance the scalability of iNM. Furthermore, by tailoring treatments to individual brain maps, than one-size-fits-all methods, we expect to achieve better results with fewer iNM treatment sessions, leading to enhanced therapeutic outcomes with greater efficacy and efficiency than non-individualized methods;
- *experimental design*: implementing an appropriate experimental design to determine the mechanisms of VP it was important to develop a task that engaged working memory, selective attention and MP networks. We thus developed a motion direction discrimination task that engaged VI in the central vision and VP in the peripheral vision. This task (electronic supplementary material, figure SI-2) required participants to: (a) discriminate up or down direction of the external stimulus’ motion presented at strong or weak-coherence levels in the peripheral vision; and (b) superimpose the peripheral stimulus’ direction of motion in the central space through VI of the internally generated percept; and
- *neurorehabilitation target and physiological response*: pair the specific function to be neurorehabilitated with the identification and elicitation of the desired physiological response. In our study, it was crucial to control the interplay between peripheral and central vision, which requires oculomotor planning along with divided attentional processes that regulate error awareness between the constantly adapting VP and VI systems. The rationale for targeting selective attention and working memory networks in our study is built upon not only behavioural studies but also molecular studies which show how visual system mechanisms are modulated through neuromodulation techniques, such as optogenetics. Targeting MP networks is based on evidence showing that saccadic eye movements are preceded by shifts in visual attention. This indicates the early engagement of top-down control mechanisms during the preparation of saccades [94–98]. In our study, engaging the MP network was crucial for switching between central and peripheral visual space, guided by selective exteroceptive and interoceptive attention (SEIA), respectively. The resulting visual spatiotemporal processes that lead to functional gains are supported by selective attention (also referred to as vigilance) through serotonergic and dopaminergic neuromodulation mechanisms, which engage early and higher visual cortex areas, thereby improving perceptual processes [99–101].

In this proof-of-concept study with healthy participants, iNM training involved VP through direction discrimination tasks at full (100%) and weak (33%) coherences and VI of motion direction. This was achieved by targeting individual networks during the presentation of a visual stimulus at central fixation space, which served as both a cue and a neuromodulation interface (figure 1*a*). We extract the maximum signal intensity time series from the control condition and boost them by lowering the signal intensity threshold in the iNM condition (figure 1*c*). Specifically, we decode the BOLD signal intensities in real-time, as subjects perform direction discrimination at full and weak coherences by increasing the difference from their control condition, i.e. increased task difficulty based on their maximal baseline time series. Decoding is done in real-time and converted into per cent change in BOLD signal intensity and spatial extents, indicated using a colour-coded representation for upregulation (red interface) and downregulation (blue interface) of the VP network. This approach allows us to guide the spatial extents and intensity of BOLD signal changes in each subject’s unique network, as a function of time. Thus, by guiding and regulating BOLD spatiotemporal modulation, iNM reduces our reliance on an individual’s ability to successfully self-regulate activity in areas that control specific physiological responses to be neuromodulated.

The goals of this study were: (i) to encode iNM-induced mechanisms that control VP; and (ii) decode [102] these spatiotemporal mechanisms to predict the probability of upward or downward motion during iNM and control conditions [103,104]. To do this, we targeted with 1 mm precision each healthy participant’s unique anatomy and strengthened the functional extents and intensity of the MT (also known as V5/MT or the hMT+ complex) that includes the MST area. The rationale to target these specific areas is based on fMRI studies [3,105] and macrostimulation studies in macaques, which showed that MT and MST signals guide visual motion perception [106–112]. We designed the iNM interface to capture and display the mean intensity and extent of BOLD signal changes (electronic supplementary material, figure SI-2) from each individual’s unique MT and MST areas (electronic supplementary material, figure SI-10). Our experimental design, based on VP learning mechanisms, effectively engaged the VP, VI, MP and selective attention neural networks, laying the foundation for future clinical applications of this intervention. Participants were asked to discriminate the direction of motion presented in their peripheral visual space and superimpose this motion direction in the central visual space, engaging the VP and VI networks, respectively. Since, VP is known to be modulated in a bottom-up mechanism, and VI in a top-down fashion [59,113–115], we hypothesized that our task would activate VP and VI networks through peripheral and central vision, respectively, by strengthening: (i) early visual areas that control VP; and (ii) higher visual, attention and working memory areas that participate in VI. The task also required the engagement of selective attention and MP networks to accurately discriminate motion direction for both full and subthreshold coherence levels. We found that the iNM strengthens VP, VI, MP and SEIA by increasing the signal magnitude in early and higher visual areas, parietal and frontal areas. Farb *et al.* [116] provide insights into how attention can be differentially modulated based on whether it is focused on external sensory input or internal bodily states, which is relevant to understanding how iNM may influence attention networks related to both exteroceptive and interoceptive processes. These networks regulate direction discrimination, with VI relying on overlapping regions associated with selective attention and working memory [117], thereby illustrating how attention modulates both VP and VI [118]. We also found that when using linear support vector machine (SVMs) to decode the direction and coherence of motion, higher classification accuracies were generated across all four networks under iNM, compared to the control condition.

This proof-of-concept study demonstrates the feasibility of iNM to enhance the BOLD signal magnitude in VP, VI, MP and SEIA networks through encoding and decoding models. We propose a safe, non-invasive framework for implementing iNM to neurorehabilitate visuospatial and low-vision deficits, specifically targeting cortical blindness and SCI, both of which currently lack effective treatments.

## 2. Material and methods

### (a) Subjects

Eight healthy, right-handed volunteers (four males, four females, age range = 25–31) were recruited into this 3-day study after obtaining informed consent according to the Baylor College of Medicine Institutional Research Board. Exclusion criteria included prior and current medical or psychiatric diagnoses, intake of any medications and general contraindications against MRI examinations. Participants had normal or corrected-to-normal visual acuity with MRI-compatible glasses. At the end of each study day, participants were compensated for their time. Although our sample size is small it did allow for thresholding at a significance level of *p* < 0.05, which was corrected for multiple comparisons (FWER-corrected = 0.01).

### (b) Magnetic resonance imaging and functional magnetic resonance imaging pulse sequence parameters

Structural and functional brain imaging was performed at the Core for Advanced Magnetic Resonance Imaging, at Baylor College of Medicine, Houston, Texas, using a 3.0 T Siemens Prisma (Siemens, Erlangen, Germany). We used a 20-channel head/neck receiver-array coil to acquire images. A T1-weighted three-dimensional magnetization-prepared, gradient-echo (MPRAGE) sequence acquired 192 high-resolution axial slices (field-of-view (FOV) = 245 mm² × 245 mm²; base resolution = 256 × 256; repetition time (TR) = 1200 ms; echo time = 2.66 ms; flip angle (FA) = 12°). Functional data consisted of 33 interleaved axial slices acquired using an Echo planar imaging (EPI) sequence (FOV = 200 mm² × 200 mm², voxel size = 3.1 mm × 3.1 mm × 3.0 mm, TR = 2000 ms; FA = 90°, number of volumes = 244).

### (c) Identification of localized networks

We acquired functional localizer scans on Day 1 to identify each participant’s individualized middle temporal and superior temporal networks that control visuospatial perception, while the participant performed the task, as described in the control condition, under "Task Design" below, §e. After scanning, the acquired anatomical and functional images were processed offline using analysis for functional neuroimages (AFNI [119]; http://afni.nimh.nih.gov/afni). The anatomical data was spatially transformed to Talairach space using an average volume of 452 skull-stripped brains (TT_icbm452; http:// www.loni.ucla.com). The functional data was preprocessed to reduce artefacts and increase signal-to-noise ratio (SNR). Our signal pre-processing protocol included: (i) removal of outliers (head motion, physiological artefacts; 3dDespike) from the time series; (ii) slice-time correction (3dTShift); (iii) transformation of our oblique-acquired functional dataset to a cardinal dataset (3dWarp -oblique2card); (iv) motion correction by registering each functional dataset to the first volume of the first functional run, using a three-dimensional rigid-body transformation; (v) spatial smoothing with a 4 mm FWHM Gaussian kernel filter; and (vi) co-registration of the functional data with the individual T1-weighted three-dimensional structural data.

The five functional localizer scans acquired on day 1 were then concatenated to increase the SNR prior to generating parametric brain maps across conditions through generalized linear models (GLM; 3dREMLfit to adjust for temporal correlations in each voxel’s time series, AFNI) for each direction and coherence. Each GLM (one for up direction and another for down direction) included: (i) four regressors, one for each of the four coherences (100%, 84%, 66% and 33% cortical direction selectivity); (ii) six covariate vectors that controlled for head motion; (iii) white matter and cerebrospinal fluid means were regressed out to increase our SNR, since activity in these areas represents noise; and (iv) the baseline-random motion direction at 0% coherence. We removed the first three TRs (6 s) from our rest-baseline condition to account for the lag of the hemodynamic response. Following a cluster analysis, using a cluster size of 20 voxels, we identified a significant network of MT and superior temporal ROIs (voxelwise *p*‐value < 0.05).

Optimization and efficacy of iNM is enhanced by carefully delineating the intensity and extent of the cortical network based on the patient’s unique anatomy during the performance of the task (electronic supplementary material, figure SI-10). Localization of each participant’s unique areas was performed by contouring the MT and MST networks associated with maximal upregulation of visuospatial task for up or down direction discrimination at 100% and 33% coherence. We find the intersection between the anatomical region and the functional intensity of each subject, while the s/he engages in the VP task prior to iNM. We then optimized each participant’s network by carefully eliminating areas that represent noise. For example, BOLD activity in MT and MST that extended only over a slice or two was eliminated.

### (d) Real-time functional magnetic resonance imaging neuromodulation acquisition

Turbo-BrainVoyager (TBV; v. 2.0; Brain Innovation, Maastricht, The Netherlands) software was used to perform the following five pre-processing computations on EPI images acquired at every TR: (i) three-dimensional motion correction; (ii) incremental linear detrending to remove BOLD signal drifts; (iii) statistical brain map displays generated from a GLM along with beta weights (BOLD signal intensity values) for each condition; (iv) extraction of average BOLD signal intensity values from individualized networks acquired on day 1 scans (see §2h*;* electronic supplementary material, figure SI-10); and (v) presentation of the network average BOLD signal intensity through the neuromodulation interface (figure 1*a*). The ***i*** ndividualized real-time *fMRI* ***N*** euro ***M*** odulation (***iNM***) interface steps are summarized in figure 1. To increase the SNR, we used an exponential moving average [120] algorithm to high-pass filter the ROI BOLD average and suppress low-frequency noise components such as scanner drifts and physiological noise effects (e.g. heart rate and respiration). The EMA output was then low-pass filtered through a Kalman filter to eliminate high-frequency noise [121,122].

### (e) Task design

A random dot kinematogram (RDK) within a circular aperture (12° radius—as described by Newsome & Paré [60] was presented to the lower right quadrant of each subject’s right visual field, while they fixated on a dot in the middle of the screen (electronic supplementary material, figure SI-2a). The RDK stimulus, generated using MATLAB (MathWorks) and Psychtoolbox [123] consisted of dark dots with a density of 2 dots/degree^2^ and a radius of 0.1°, and presented on a light grey background to minimize scattering. The RDK displayed upward or downward motion at four levels of coherent motion: 100%, 84%, 66% and 33% indicating the percentage of dots moving in the same direction. A random fraction of the dots was displaced by 0.2 in up or down direction at a rate of 4° s^−1^, while the rest were replaced by new dots at random positions. At coherence levels of 84%, 66% and 33%, the random dot pattern was refreshed every 50 ms. Each run followed a temporal sequence with a 20 s coherent motion block interleaved with a 10 s baseline-random motion block at 0% coherence.

Here, we focus on the fully and subthreshold coherence levels represented as C100 and C33 throughout this article. Using their central vision to fixate on a dot in the middle of the screen, participants were asked to track the direction of RDK motion, which was presented in the lower quadrant of their right visual field, through their peripheral vision as it alternated between up or down direction versus random motion. We had dedicated scans that presented motion in the up direction and separate scans that presented motion in the down direction. The coherence levels were randomly counterbalanced across scans to ensure a balanced and unbiased presentation of stimulus. Owing to cost constraints and limited subject availability for extensive psychophysics assessments, we set a subthreshold limit of 33%. This decision is supported by Ward *et al.* [124], who report that above this threshold, it is expected that all subjects can perform the task. All our subjects indicated that the task was very difficult at 33% coherence; i.e. on a scale of 1–10 (0: can immediately detect the direction of motion; 10: cannot detect the direction of motion), they rated the task a 7–8.5 (up direction) and 9–9.5 (down direction) in terms of difficulty to discriminate motion direction.

In the control and neuromodulation conditions, participants were asked to use their VI by superimposing the upward or downward direction of motion centrally, while the direction of motion was tracked through their peripheral vision, since the RDK was presented in their peripheral visual space. We asked participants to visualize the 64 feet tall Texas Medical Center Waterfall, which is located just a few feet away (across the street) from the Papageorgiou Laboratory. All our participants were familiar with the waterfall since they were students at Baylor College of Medicine and viewed it daily. The waterfall droplets travel in downward and upward direction. Thus, the mental imagery we coached participants to adopt accounted for any confounding factors as it encompasses the motion direction and size of the droplets’ components. We specifically chose this mental imagery because it closely resembles the motion of the RDKs.

In the iNM condition, the central dot served as the neuromodulation interface, which enforced eye fixation in the middle of the screen to avoid non-purposeful eye movements which would result in BOLD activity unrelated to the requested task: (i) when the central dot filled with red colour, it corresponded to successful VP and VI , which translated to upregulation of the BOLD intensity within the individualized networks we targeted during iNM that selected for upward or downward direction of motion; and (ii) when the central dot filled with blue colour, it corresponded to successful VP and VI , which translated to downregulation (inhibition) of the BOLD intensity within the individualized networks we targeted during iNM that selected for upward or downward direction of motion. The direction of motion was interleaved with blocks of random motion, during which subjects were asked to rest by disengaging from superimposing imagery of direction of motion, while continuing to fixate on the central dot, which also served as the iNM interface.

### (f) Study structure

Our study included two sessions: each consisting of 10 functional (also called EPI) scans, which included five control—no iNM scans that alternated with five neuromodulation (iNM) scans. Each EPI scan included eight continuous periods each lasting 8 min and 12 s. Within each period, subjects were cued to imagine motion perception as either up or down depending on the RDK session displaying one of the four coherence levels. Each coherent motion block lasted 20 s and was interleaved with a baseline-random motion block (10 s). The direction of coherent motion blocks was randomly counterbalanced across runs, following three rules: (i) each coherent motion block occurred twice during each period; (ii) a coherent motion block was never followed by the same coherence level; and (iii) each run consisted of a unique block order.

### (g) Neuromodulation

Neuromodulation was determined by colour and extent of a circle that was filled, representing the magnitude and extent of each subject’s targeted network. The neuromodulation signal was calculated by comparing the per cent BOLD signal change (PSC) generated during each iNM run for each direction and coherence block with the corresponding direction and coherence in the control condition. The BOLD PSC change was calculated from each participant’s individualized areas every 2 s as follows:


BOLDPSCi(j)=100%∗[ROIsBOLDduringUpORDowndirectionandcoherencei(j)−ROIsBOLDduringcorrespondingcontroldirectionandcoherence,i(j)]/randommotion−resti(j),

where *i* represents the coherence level (C100, C84, C66 and C33) and *j* represents the 2 s time interval used to compute the BOLD PSC at each coherence level. To determine whether the BOLD was upregulated or downregulated, the iNM condition was compared to the control using a 10th percentile threshold. The 10th percentile was calculated from the overall BOLD signal change during the control condition that preceded the iNM run. If the PSC during a 2 s interval exceeded the 10th percentile of the control condition, the iNM interface indicated a 10% upregulation. Conversely, if the signal was below the 10th percentile, it indicated downregulation. The neuromodulation presented at each TR was computed by comparing the PSC value generated every 2 s with reference range, which was derived from the comparison of iNM BOLD signal intensity to the control condition (figure 1*c*). The reference range included eight bins of 25% BOLD increase or decrease: −100%, −75%, −50%, −25%, 0, 25%, 50%, 75% and 100%. During the iNM run following each control run, if the PSC at a given time point was within or exceeded the threshold value of the reference range, it was indicated by filling the circle with red. This represented the upregulation of the targeted individualized ROI BOLD signal that was activated in response to the VP–VI task. If the PSC was lower than the minimal value, the circle was filled with blue, representing the downregulation of the targeted ROI BOLD signal that controlled the VP–VI networks.

### (h) Analysis

Acquired anatomical and functional images were processed offline using AFNI [119]; http://afni.nimh.nih.gov/afni). Anatomical data was spatially transformed to Talairach space using the TT_N27 atlas. Functional data was preprocessed to reduce artefacts and increase SNR. Our signal pre-processing protocol included: (i) removal of outliers (head motion, physiological artefacts; 3dDespike0 from the time series); (ii) slice-time correction (3dTShift); (iii) transformation of our oblique-acquired functional dataset to a cardinal dataset (3dWarp -oblique2card); (iv) motion correction by registering each functional dataset to the first volume of the first functional run, using a three-dimensional rigid body transformation; (v) spatial smoothing with a 6 mm FWHM Gaussian kernel filter; (vi) co-registration of functional data with individual T1-weighted three dimensional structural data; and (vii) scaled to have a mean BOLD signal intensity of 100.

Statistical analysis was performed on fMRI data using a GLM approach. Five runs for each condition (i.e. control or iNM) were then concatenated to increase the SNR prior to generating parametric brain maps across different coherence levels for each patient. A second-order polynomial was used to model slow baseline shift. Model parameters were estimated using the 3dREMLfit program in AFNI, which uses an ARMA(1,1) model to estimate the correlation structure in noise. The GLM included: (i) four regressors, one for each of the four coherence levels (C100, C84, C66 and C33); (ii) six covariate vectors that controlled for head motion; (iii) regression of white matter and cerebrospinal fluid means to increase SNR, since activity in these areas represents noise; and (iv) baseline-random motion blocks. The four regressors corresponded to each coherence level and were convolved with a gamma variate function, a canonical hemodynamic response function. Group analysis was then performed using 3dMEMA, a mixed effects meta-analysis program that models within and across subject variability. After generating a network of regions for VP–VI control compared to rest-baseline (voxelwise *p*‐value = 0.05), cluster analysis was performed to identify highly significant ROIs (FWER < 0.01) using a cluster size of at least 10 voxels (3dClustSim, AFNI).

The remaining analysis was performed in Python. We focus here on the analysis for coherence levels C100 and C33. Significant ROIs for each coherence level and motion direction were analysed and certain ROI clusters that represented dataset noise were removed. Anatomical masks as defined in the AFNI TT_Daemon atlas were created and resampled for alignment with participants’ functional scans. These functional masks created after day 1 from the localizer scans were then used to extract statistically significant ROIs that corresponded to the MT and MST cortices. Functional data was detrended using a second-order polynomial before being scaled to a mean BOLD signal intensity of 100. Time series BOLD responses were extracted across the entire brain for analysis to generate significant networks formed from the control and iNM conditions. Extracted time series signals for specific brain regions were used to conduct the remaining analyses.

### (i) Networks

We designed a task that would capture the visuospatial networks needed to perform a visuospatial task. The task activated four distinct networks: VP, VI, MP and SEIA. The individual brain regions contained within each network are listed in the electronic supplementary material, figure S1.

### (j) Support vector machine classification

A linear SVM was used to decode BOLD spatial patterns of each coherence level (i.e. C100 and C33) generated by the five neuromodulation and five control conditions. Each functional run included 120 three-dimensional participant fMRI scans representing the BOLD signal at a particular TR (120 TRs total). Within each run, the selected coherence level was labelled 1 and the random motion block following the selected coherence level was labelled 0. If the selected coherent motion block was at the end of the run, the preceding random motion block was used as the baseline and labelled 0. The SVM was trained to distinguish between these two classes for each coherence level. In our study, we aimed to develop a model that is both robust and generalizable across varied sources. To achieve this, we integrated different coherence levels, directions, networks and conditions. We then employed a comprehensive fivefold cross-validation technique, a common and effective strategy to optimize hyperparameters like C in SVM analysis and tested values ranging from 10^−5^ to 10^5^ to ensure that our model found the optimal balance between bias and variance. The primary metric for assessing model performance was the area under the receiver operating characteristic (AUROC) on each validation set [125]. The optimal C value was identified as the one that provided the highest mean AUROC across all validation folds. This strategy of integrating data across coherence levels, directions and conditions and applying stringent cross-validation criteria accounts for potential overfitting and bias, 2006). It ensures that our model is not excessively tailored to specific subsets of data (coherences versus directions versus networks versus conditions) and maintains high performance across all examined scenarios. For each subject and each coherence level, we trained the SVM model on the concatenated data from four runs of the corresponding condition (i.e. control or iNM) and tested its performance using AUROC on the remaining run. This was repeated in a cross-validation approach and the AUROC’s were averaged to achieve a single AUROC for each subject with a coherence level. Differences in classification accuracies were compared across coherence level conditions and a paired *t*‐test was run using the AUROC values for each subject. **SVM was also used to decode BOLD spatial patterns within predefined networks (VP, VI, MP, SEIA) for each coherence level.**

### (k) Area under the curve (AUC)

To compute the BOLD PSC for each coherence level and direction of motion as a function of time, ROIs were categorized across VP, VI, MP and SEIA networks. BOLD PSC time series were generated by averaging: (i) the time series of voxels within an ROI; and (ii) over signal time segments from blocks of the same coherence level collected from runs of the same direction. Each signal segment started at the onset of a coherent motion block and ended at the last time point of the following random motion block with the exception of the last block of the scan, which used the random motion block preceding the coherent motion block, since the scan ended with a coherent motion block. Each subject had five control and five iNM runs with each run entailing two blocks of VP–VI at each coherence level. The PSC time series for each coherence level, each condition and each subject was averaged over 10 BOLD signal segments. The area under the PSC curve of each condition (control or iNM) was calculated using Simpson’s rule:


$$AUC_{con}^{reg}=\frac{1}{3}(PSC_{1}+4PSC_{2}+2PSC_{3}+\ldots +4PSC_{T-1}+PSC_{T}$$

where PSC_*t*_ is the PSC at the corresponding time *t*.

The BOLD PSC was then calculated using the following formula:


$$100*\left({AUC}_{cNMT}^{reg}-AUC_{ctrl}^{reg}\right)/AUC_{cNMT}^{reg}.$$

### (l) *D*’ sensitivity index

To measure the strength of iNM across time and rank regions, we used a variant of Cohen’s *d* known as the *D*’ sensitivity index. This index quantifies the separation between the control and iNM distributions means relative to their standard deviations. Assuming that the fMRI dataset has T time points, B blocks, S subjects and N ROIs, each ROI was characterized by a three-dimensional matrix of dimensions T×B×S . Each subject was then characterized by a two-dimensional matrix of dimensions T×B . For a fixed time point t , the distributions of control and iNM blocks were $dist_{ctrl}^{s}\left(t\right)$ and $dist_{nFb}^{s}\left(t\right)$ , respectively. *D*’ for the subject *s* at time point t is:


$$d^{s\prime }\left(t\right)=\frac{\mu_{iNM}^{s}\left(t\right)-\mu_{ctrl}^{s}\left(t\right)}{\sqrt{\frac{1}{2}\left(\sigma_{iNM}^{s}{\left(t\right)}^{2}+\sigma_{ctrl}^{s}{\left(t\right)}^{2}\right)}},\;t=1,\ldots ,T$$

where, $\mu_{con}^{s}\left(t\right)=mean\left({dist}_{con}^{s}\left(t\right)\right),\sigma_{con}^{s}\left(t\right)=std\left({dist}_{con}^{s}\left(t\right)\right).$

The sensitivity index between the two conditions at time point t and for the fixed region is the average sensitivity index across the subjects:


$$d^{\prime }(t)=\underset{s}{mean}\left(d^{s\prime }\right),s=1,...,s.$$

## 3. Results

### (a) Decoding: individualized neuromodulation (iNM) improves classification performance over control—no individualized neuromodulation condition using linear support vector machine analysis

To determine if iNM strengthens the VP and VI networks, we analysed direction discrimination responses under control and iNM conditions using encoding and decoding at two coherence levels: fully coherent (100%) and subthreshold coherence (33%). Similar to brain stimulation interventions, neurofeedback studies can establish causal links between brain activity and physiological responses (VP and VI in this study), rather than just correlations. For example, fMRI neurofeedback can endogenously manipulate brain activity as an independent variable, thus, altering the brain network [126–132]. Thus, by using encoding and decoding models, we gained insights into the causal relationships of iNM-induced mechanisms. Specifically, we: (i) quantified how the brain encodes motion direction and coherence; and (ii) decoded brain activity to predict accuracy of motion direction and coherence under control and iNM conditions.

**Figure 2.**
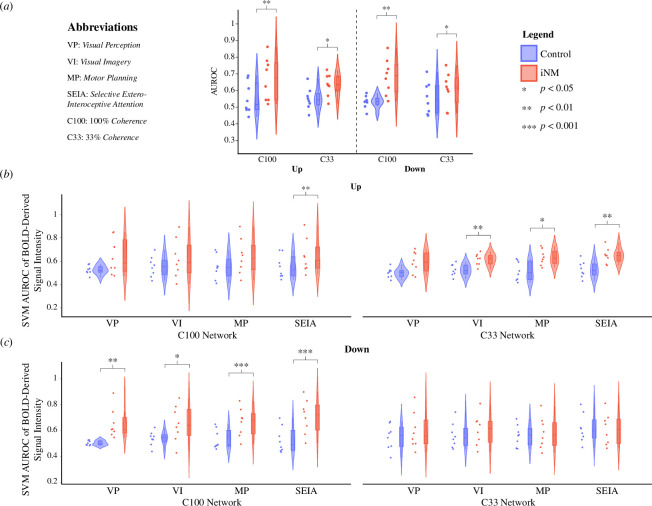
Increased classification performance in iNM condition over control—no iNM condition for all coherence levels and networks using a linear SVM. (*a*) Violin plots of SVM-generated AUROC (*y*-axis) performance values across all subjects relative to coherence level (*x*-axis) and training-testing data type permutations (colour coded). On each box, the central line and lower and upper edges represent the median (Q2), 25th (Q1) and 75th (Q3) percentiles, respectively. The dots represent outliers (bigger than Q3 by 1.5*(Q3 - Q1) or smaller than Q1 by 1.5*(Q3 - Q1)). Whiskers extend to the most extreme non-outlier datapoints. Black lines and asterisks indicate significant differences by paired-samples Wilcoxon sign tests. (*b*) Violin plots of SVM-generated AUROC performance values across all subjects relative to C100 and C33 coherence levels in up direction cortical networks. (*c*) Violin plots of SVM-generated AUROC performance values across all subjects relative to cortical networks in C100 and C33 down direction coherence levels.

To assess brain changes associated with motion direction and coherence under iNM and control conditions, we used a linear SVM and trained decoder models within each network (figure 2*b,c*) and across (figure 2*a*) networks to predict the motion direction and coherence of visual stimuli (see §2 for details). We found that a linear SVM could significantly improve classification performance under neuromodulation as shown by the median AUROC under all coherence levels and motion directions. The median AUROC for each coherence level and motion direction was: (i) 0.68 AUROC iNM versus 0.52 AUROC control for **C100 up** (*p*‐value: 0.001); (ii) 0.71 AUROC iNM versus 0.53 AUROC control for **C100 down** (*p*‐value: 0.004); (iii) 64 AUROC iNM versus 0.54 AUROC control (*p*‐value: 0.045) for **C33 up**; and (iv) 0.63 AUROC iNM versus 0.55 AUROC control (*p*‐value: 0.048) for **C33 down** (figure 2 *a*; electronic supplementary material, table S5a).

When we tested the effect of including VP, VI, MP and SEIA networks within the classification scheme (figure 2*b,c*; electronic supplementary material, table S5b), the classification performance of two networks in the C100 up condition was higher under neuromodulation than in the control condition: MP network (0.63 AUROC iNM versus 0.55 AUROC control; *p*‐value: 0.05) and SEIA network (0.61 AUROC iNM versus 0.53 AUROC control; *p*‐value: 0.004). The remaining two VP, VI C100 up networks were trending towards significance but did not achieve it: VP (0.58 AUROC iNM versus 0.55 AUROC control; *p*‐value: 0.06) and VI (0.59 AUROC iNM versus 0.56 AUROC control; *p*‐value: 0.08). In the C33 up condition, three networks achieved significance: VI (0.59 AUROC iNM versus 0.51 AUROC control; *p*‐value: 0.07), MP (0.63 AUROC iNM versus 0.50 AUROC control; *p*‐value: 0.02) and (0.65 AUROC iNM versus 0.52 AUROC control; *p*‐value: 0.004). The VP network for C33 was trending towards significance but did not achieve it (0.59 AUROC iNM versus 0.51 AUROC control; *p*‐value: 0.07). All C100 down networks were statistically significant: VP (0.61 AUROC iNM versus 0.50 AUROC control; *p*‐value: 0.006), VI (0.64 AUROC iNM versus 0.54 AUROC control; *p*‐value: 0.02), MP (0.68 AUROC iNM versus 0.49 AUROC control; *p*‐value: 0.0007) and SEIA (0.72 AUROC iNM versus 0.49 AUROC control; *p*‐value: 0.0008). No networks in the C33 down condition achieved or trended towards significance: VP (0.56 AUROC iNM versus 0.56 AUROC control; *p*‐value: 0.32), VI (0.62 AUROC iNM versus 0.54 AUROC control; *p*‐value: 0.29), MP (0.57 AUROC iNM versus 0.56 AUROC control; *p*‐value: 0.32) and SEIA (0.61 AUROC iNM versus 0.60 AUROC control; *p*‐value: 0.86).

### (b) Encoding: individualized neuromodulation increased mean area under the curve signal intensity and *d* prime (*d*’) sensitivity indices compared to control conditions across networks, motion directions and coherence levels

**Figure 3.**
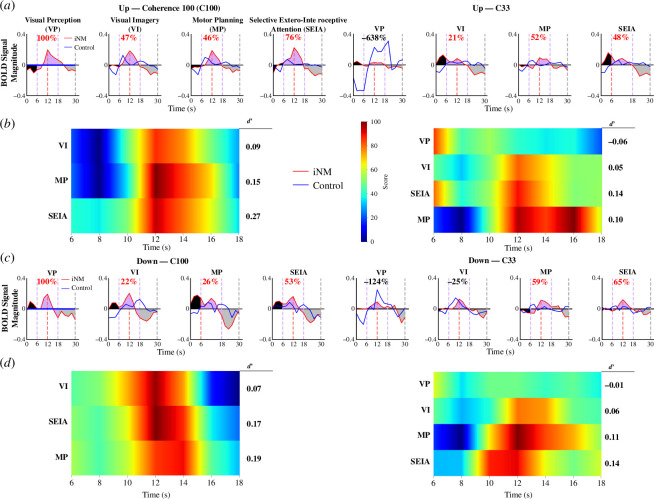
iNM increases mean AUC signal intensity and *d*’ sensitivity indices compared to control conditions across networks, motion directions and coherence levels.(*a*) BOLD signal intensity of both iNM and control conditions are plotted as a function of time for all networks and coherence levels in the up-motion direction. Each plot contains three sections: (i) a haemodynamic lag block (0–6 s); (ii) a coherent motion block, which was truncated to 18 s owing to participant fatigue in the task, as the signal decreased across all ROIs (6–18 s); and (iii) a baseline-random motion block (18–30 s). The area under the BOLD signal intensity curve of the iNM condition is coloured black to indicate the haemodynamic lag; purple for coherent motion; and grey for baseline-random motion blocks. Per cent change in AUC between iNM and control conditions for the coherent motion block is in red. (*b*) The *d*’ sensitivity index plotted as a function of time in the coherent motion block for all networks and coherences in the up-motion direction. The *d*’ sensitivity value for the entire period is shown to the right of each heatmap. (*c*) BOLD signal intensity of iNM and control conditions plotted as a function of time for all networks and coherence levels in the down-motion direction. (*d*) The *d*’ sensitivity index plotted as a function of time in the coherent motion block for all networks and coherences in the down-motion direction.

The per cent change in AUC signal intensity illustrates the effect of neuromodulation on signal intensity over the course of each block in the scan (figure 3). The effect of neuromodulation was greater on VP and VI networks. The VP network was significantly higher by 100% for both directions at C100 coherence, but lower at coherence level C33, with a −1362% change in AUC signal intensity for the up direction and −124% for the down direction. The VI network was also higher by 47% in the up direction and 22% in the down direction at C100 coherence. At coherence level C33, the VI network increased by 21% in the up direction but decreased by 25% in the down direction. *Interestingly, at coherence level C100, no significant VP network activity was seen in either direction in the control condition*, *with significant VP network activity only seen during iNM.* The MP network was upregulated at all coherence levels and directions in the iNM condition compared to the control with AUC signal intensity ranging from 26% to 59% across directions and coherences. iNM SEIA networks also increased with AUC signal intensity ranging from 48% to 76% across directions and coherences. Detailed analyses of each ROI for each network across all coherence levels and directions are shown in the electronic supplementary material, figures SI-3**–**SI-8).

The *d*’ sensitivity index was also computed for each experimental time point in the form of heatmaps. The *d*’ sensitivity index measures the separation between the means of the control and iNM distributions relative to their standard deviations. For the C100 up condition, all three networks (VI, MP and SEIA) had similar *d*’ sensitivity index profiles, with greater activation in the control condition after 10 s, at which time activation under the neuromodulation condition increased above the neuromodulation condition. For the C33 up condition, the VP network peaked first, followed by peak activation of VI and SEIA around 12 s and finally MP at 16 s. Activation of all networks in the C100 down condition peaked at 12 s; however, activation of the VI network started earlier than in the SEIA and MP networks and also began to ramp down earlier. Activation of SEIA and MP lasted longer (until 15 s). Finally, in the C33 down condition, VP was activated first followed by the SEIA network and simultaneous peak activation of the VI and MP networks. However, activation of the MP network began earlier, near the start of SEIA network activation.

## 4. Discussion

The purpose of this proof-of-concept study was to determine whether iNM could strengthen early and higher VP, VI, MP and SEIA networks. By applying encoding and decoding models, we were able to move beyond simple correlations and uncover the underlying spatiotemporal mechanisms. Encoding predicts brain activity from stimuli, while decoding, extracts information about the stimuli from brain activity.

VP is the result of the interaction between ongoing spontaneous activity in the visual cortex and the activity evoked by a stimulus [133]. *The VP task engaged the peripheral visual field* to perceive randomly moving dots at strong (100%) and weak (33%) coherences. By contrast, *the VI task engaged the central visual field* by superimposing the mental imagery (internally generated) stimulus at central eye fixation, where the iNM interface was located (figure 1*a*). We quantified the spatiotemporal evolution of BOLD magnitude using two metrics: (i) an SNR sensitivity index (*d*’); and (ii) the AUC. Compared to the control condition, iNM generated significantly higher *d*’ and AUC values under strong (100%) coherence in the VP network (100% increase for both directions) and in the VI network (47% for upward and 22% for downward directions). However, at weak, subthreshold (33%) levels, iNM reduced the AUC of the BOLD magnitude in the VP network in both directions. By contrast, iNM increased the AUC of the BOLD magnitude in the VI network in the up direction by 21% but decreased it in the down direction by 25% (figure 3).

All participants reported that the VI of downward motion was much harder than upward motion imagery during both the control and iNM conditions. This suggests a challenge in upregulating the VI network during downward motion imagery, consistent with participants’ unanimous reports that downward motion was harder to imagine, aligning with participants' difficulty performing the task. The lack of increased VP when motion coherence (the consistency of motion direction in the stimulus) is weak under iNM, compared to the control condition could be attributed to several factors. One hypothesis is that the interaction between ongoing activity in the early visual cortex and stimulus-evoked activity creates a bottleneck, necessitating the allocation of perceptual resources to the iNM interface cues. Previous studies have shown that VP relies on interactions between visual cortex activity and characteristics of the stimulus [134].

Another plausible hypothesis is that although iNM decreased the BOLD magnitude in VP areas, it simultaneously increased the magnitude in the VI, MP and SEIA networks, as the task involves processing both externally presented stimuli in the peripheral visual space and internally generated (mental) imagery in the central visual space. This suggests that a balanced, redistributed allocation of resources is essential to successfully meet task demands under iNM. iNM increased the BOLD magnitude in the MP (up: 52%, down: 59%) and SEIA (up: 48%, down: 65%) networks, suggesting that effectively managing the physiological demands of this complex VI task requires engaging working memory to generate internal visual images, along with SEIA and MP processes. Consistent with previous reports [76,135–138], continuous communication between bottom-up and top-down control networks is necessary, where VP operates as a bottom-up mechanism and VI as a top-down mechanism. This interpretation aligns with microstimulation studies on direction discrimination tasks involving weak-coherence stimuli, which were not reliably discriminated [108,109]. It has been shown that the extent and intensity of visual cortex activity during stimulus presentation depend on specific characteristics, such as contrast sensitivity—the difference in luminance or colour that makes an object distinguishable from others—[134], where higher coherence (100%) results in stronger and more easily detectable signals in the visual cortex.

We found that modulating visual sensory direction and magnitude of coherence inputs interpreted by the VP and VI networks strengthen areas involved in the crosstalk between selective exteroceptive attention (interpreted by the VP network) and interoceptive attention (interpreted by the SEIA network) in the iNM. This crosstalk between the two attention systems controls conscious perception and awareness of one’s errors [139]. Attention can be driven exogenously or endogenously [140,141], with selective attention divided into focused and divided attention. In this study, exteroceptive attention was driven exogenously by incoming peripheral sensory information through the VP network, while interoceptive attention was driven endogenously by internally generated (mental) imagery of the stimulus. Thus, selective attention was divided, as these two systems constantly control the interplay between exteroceptive (VP) and interoceptive (VI) attention. iNM enhanced the BOLD intensity in areas responsible for selective attention, involving communication between: (i) exteroceptive attention through incoming peripheral visual sensory information, that activates visual motion perception areas; and (ii) interoceptive attention focused on internally generated (mental) images of up or down directions at full and subthreshold levels.

The MP and SEIA networks exhibited an increased AUC of BOLD magnitude under the iNM condition across all directions and coherences. Activity within MP areas appears to be associated with oculomotor planning and control, specifically between tracking the VP stimulus in the peripheral space and generating mental imagery of the stimulus at central eye fixation. Common MP areas, including the precentral–primary motor area—known to respond to visual stimuli during the planning and initiation of movement—and the anterior motor cerebellum, particularly the fastigial nucleus, play crucial roles in sustaining persistent preparatory control [142].

The cerebellum, which is well-established in controlling eye movements and visually guided motor learning, influences perception and motor control by modulating projections to sensorimotor areas such as the premotor cortex, supplementary motor area and precentral and primary somatosensory cortex [143–145]. There are various hypotheses regarding cerebellar processing, including forward and inverse internal models. The forward model predicts oculomotor position, while the inverse model plans appropriate eye movements between peripheral and central visual spaces according to task demands [146]. These models offer complementary or synergistic perspectives on interpreting activity from oculomotor planning and control areas. Our results suggest that persistent neural dynamics during MP are sustained by multi-regional neural circuits, such as the anterior motor cerebellum, which is crucial for online motor control dictated by task demands [147,148]. The recruitment of anterior motor and posterior sensory cerebellar regions [148] is also required as successful VP relies on the interplay between ongoing spontaneous visual cortex activity and that evoked by a stimulus [133].

The precentral–primary motor area, along with the basal ganglia and supramarginal gyrus, has been implicated in higher motor cognition and the voluntary and controlled generation of visual sensory and motor imagery [149,150]. Our findings align with recent studies showing that motor and somatosensory areas are crucial in predicting oculomotor control within early visual areas, underscoring the integration of sensory and motor systems in complex visual processing [151–153].

The basal ganglia, in particular, play a key role in voluntary saccadic eye movements, transmitting inhibitory signals from the caudate to the substantia nigra and subsequently to the superior colliculus [154]. Studies in macaques have demonstrated that the basal ganglia modify oculomotor control in response to reinforcement stimuli [155]. In our study, iNM served as a reinforcement mechanism, leading to increased signal intensity in the caudate, putamen and globus pallidus compared to the control condition. Specifically, at full threshold (100%) coherence, iNM increased the BOLD magnitude in areas such as the right caudate (77% AUC increase), left caudate (81% AUC increase), right putamen (35% AUC increase) and left putamen (72% AUC increase) for the up direction. Additionally, the left putamen showed a 100% AUC increase for the down direction. At subthreshold (33%) coherence, iNM increased the BOLD magnitude in the right caudate and putamen by 100% AUC for the up direction and similarly in the right caudate for the down direction.

Our goal was to create a task ecosystem that simulates real-world deficits, enabling individuals with neurological impairments to practice relevant strategies in a controlled setting, to modify underlying neural mechanisms. We agree with [82] that fMRI interfaces should create contexts that mirror real-world scenarios where deficits typically manifest. This approach allows individuals to practise strategies safely and effectively. By simulating real environments to provoke dysfunctional neural mechanisms, the neuromodulation interface will help recruit and alter impaired brain functions. In our study, we designed an interface and task to assess visual perception by engaging spatial perception mechanisms, critical for real-world navigation. By stimulating VP, VI, MP and SEIA networks, we aimed to simulate the functions necessary for navigation in everyday life. This approach allowed us to test the interface feasibility and understand the underlying spatiotemporal mechanisms before applying the iNM interface to patients with cortical blindness and SCI. Our goal is to pave the way for more precise and personalized neuromodulation treatments.

## 5. Conclusions

### (a) Individualized neuromodulation strengthens neural networks

This proof-of-concept study demonstrates that iNM effectively enhances the activity within VP, VI, MP and SEIA networks, providing a promising approach for neurorehabilitation in conditions associated with visuospatial and low-vision deficits. iNM enhanced the AUC of the BOLD magnitude in the VP and VI networks by increasing the activity of attention and memory-related signals, in areas such as the hippocampus, frontal, intraparietal sulcus, parietal lobule and superior parietal areas. Similar findings have been reported in macaque studies [112,156,157]. iNM enhanced the AUC of the BOLD magnitude in the SEIA network across motion directions and coherences by engaging sensory posterior cerebellar (pCb) areas (electronic supplementary material, figure SI-5–7), an area known to participate in sensory, attention and working memory demands [116,158–160]. It also enhanced the MP networks. These findings align with the involvement of the dorsal attention network, which includes the pCb, parietal areas and visual motion areas, such as frontal eye fields and premotor cortex [157].

### (b) Balanced resource allocation

Under control conditions with weak coherence, the brain shows increased VP demands (AUC of the BOLD magnitude) for motion direction interpretation compared to the iNM condition. This increased activity in the VP network suggests that motion awareness and perception are more demanding, while the decreased AUC of the BOLD magnitude in the VI, MP and SEIA networks indicates that resources for imagery, MP and selective attention are less necessary under control than iNM conditions. Consequently, when coherence was weak, the BOLD magnitude in the VP network decreased under iNM compared to the control, while resource allocation appears to have been redistributed to the MP and SEIA networks. This redistribution probably facilitated adaptive monitoring of oculomotor and attentional demands between peripheral vision and central vision, performed by the VP and VI networks, respectively, in response to iNM demands. These findings underscore the importance of balanced and redistributed neural resource allocation across networks to effectively meet task demands, particularly when processing both externally presented stimuli (VP) and internally generated imagery (VI).

### (c) Uncovering spatiotemporal mechanisms

By applying encoding and decoding models, the study was able to move beyond simple correlations to uncover the underlying *spatiotemporal mechanisms* governing these neural networks, offering new insights into VP and VI networks under iNM. It is important to note that we intentionally chose the term ‘neuromodulation’ instead of ‘neurostimulation’, as iNM involves non-invasively guiding BOLD intensity and its spatial extent over time, while also incorporating elements of self-regulation. To clarify this distinction, ‘neuromodulation’ accurately reflects the combined aspects of non-invasive guidance and self-regulation inherent in our study. In summary, iNM targets specific individualized anatomical and functional networks by identifying time series with maximal BOLD intensity during the control condition and enhancing these by increasing their difference from the control condition.

### (d) Future applications

This proof-of-concept study demonstrates that iNM can effectively enhance the activity within VP, VI, MP and SEIA networks, providing a promising approach for neurorehabilitation in conditions associated with visuospatial and low-vision deficits. Our future goal is to apply this visuospatial iNM approach to neurorehabilitate low-vision deficits in cortically blind patients with early or advanced VP lesions, and to slow the progression of visuospatial deficits in patients with prodromal SCI, as no effective, safe and non-invasive intervention currently exists.

## 6. Limitations of present proof-of-concept study

To ensure rigorous analysis, we developed a computational pipeline that extends beyond simple correlations, enabling causal inferences through encoding and decoding analyses [161]. Encoding analysis quantified how the brain encodes spatiotemporal features of stimulus direction and coherence across networks, while decoding analysis predicted stimuli from brain activity maps. The statistically significant differences in AUC of BOLD magnitude across networks and directions highlight the robustness of iNM (figure 2; electronic supplementary material, figures SI-3 and SI-4). At subthreshold coherence levels, iNM reduced AUC values in the VP network for both directions and in the VI network for the down direction only, compared to the control condition. Our decoding analysis showed that iNM significantly improved classification performance through a linear SVM, as indicated by the median AUROC across all coherence levels, motion directions and networks (figure 2).

Although we did not have access to an MR compatible eye tracker to measure eye movement owing to prohibitive costs, we strategically placed the iNM interface centrally so that participants' eyes would fixate on the central visual field and we tracked motion presented in the peripheral space. If participants had difficulty maintaining central fixation, it would have affected their iNM feedback, which researchers could detect through the time series' evolution every 2 s, eventually leading to a plateau. Despite the lack of a direct behavioural measure to determine participants' direction discrimination, we assessed performance using computational models that quantified the encoding networks generated (figure 2; electronic supplementary material, figure SI-5*a*,*b*) and decoded the prediction of performance (figure 3; electronic supplementary material, figures SI-3 and SI-4). These computational models consistently agreed in their findings across direction, coherence and network.

## Data Availability

Our data and analysis resides on the Dryad repository at [162]. Supplementary material is available online [163].
